# Trends of congenital hypothyroidism and inborn errors of metabolism in Pakistan

**DOI:** 10.1186/s13023-020-01602-6

**Published:** 2020-11-14

**Authors:** Sumreena Mansoor

**Affiliations:** Department of Biochemistry, Shifa College of Medicine, Shifa Tameer-e-millat university, H-8/4, Islamabad, Pakistan

**Keywords:** Metabolic disorders, Inborn errors of metabolism, Challenges, Pakistan

## Abstract

**Background:**

Metabolic disorders are heterogeneous group of genetic disorders that are responsible for significant neonatal and infant morbidity and mortality worldwide. In developing countries like Pakistan where infant mortality is high current population based studies are unable to gauge contribution of metabolic disorders in causing mortality and morbidity. It is essential to address this gap by a review of available scattered Pakistani data related to metabolic disorders specifically congenital hypothyroidism and inborn error of metabolism to calculate probable burden of these disorders.

**Main body:**

Unfortunately currently in Pakistan newborn screening which identifies these illnesses at birth as a preventive strategy are not available. For current review data was collected through a systematic search of published articles (including data related to screening in certain subgroups of patients admitted to pediatric/neonatal intensive care units, patients with developmental delay/mental retardation).

**Conclusion:**

The primary aim of this review was to get an estimate of the disease burden in the Pakistani population as true prevalence of Congenital Hypothyroidism and Inborn Errors of Metabolism in Pakistan is not available. This systematic review will help us to identify the rough idea about the scale of problem in Pakistan.

## Background

Metabolic disorders like congenital hypothyroidism (CH) and inborn errors of metabolism (IEM) are considerable cause of disease and death among children in both the advance and developing nations of the world with the prevalence of CH is about 1 per 3000–4000 and IEM is 1 in 800–2500 births in West [1, 2]. Metabolic disorders are genetically inherited biochemical disorders of specific enzymes or proteins causing a block in a normal metabolic process of protein, carbohydrate or fat metabolism. Classification is challenging as it can occur in every biochemical pathway. Based on pathophysiology, there are three main sub groups of IEM; conditions that cause intoxication, conditions of energy metabolism and conditions of complex molecules [3]. Severity of the symptoms experienced by affected individuals of IEM varies relying on the genetic and mutation history [4]. Due to absence of neonatal screening facilities in Pakistan children born with IEMs are usually diagnosed on the basis of clinical symptoms including lethargy, poor feeding, apnea or tachypnea, and recurrent vomiting. That makes it difficult to manage childhood morbidity and mortality due to these disorders in an effective manner. Multiple studies in selected scattered groups of Pakistani patients have confirmed the high prevalence of IEMs is due to the high rate of inter-marriages and large family sizes [5]. To classify CH we can use etiological basis of disease according to which there are two main categories of these disorders (a) disorders of thyroid gland development (Group of dysembriogenesis or thyroid dysgenesis) or (b) defects in any of the steps of thyroid hormone synthesis (group of dyshormonogenesis) [6, 7].

## Status of IEM diagnosis and management in Pakistan

Though some information regarding mental retardation and new born screening (NBS) for CH in Pakistani population is available since 1987, but a true prevalence of IEM is yet to be determined [5, 8]. Relative or true incidence of IEM in the population or even among the critically ill newborns through systematic reviews is lacking as NBS is not available in Pakistan. Mostly IEM is diagnosed either through biochemical tests on specific population mostly pediatric population. Mostly studies have bias of screening for IEM in high risk patients either in intensive care of pediatrics or medicine [9].

To determine true prevalence of IEM in Pakistan one important and ideal strategy needs to be applied, i.e., Prospective data collection from a universal and expanded screening of a low risk population. Mass spectrometry is considered to be ideal for screening metabolic IEM but very few centers in Pakistan are offering these services either in collaboration with tertiary care hospitals of Pakistan or abroad for few large cities [5, 10, 11].

## Material and methods

The purpose of this study was to learn about the various forms of metabolic disorders across Pakistani population. For current review data was collected through a systematic search of published articles on metabolic disorders including congenital hypothyroidism and inborn error of metabolism among Pakistani population. Data related to screening in certain subgroups of patients admitted to pediatric/neonatal intensive care units, patients with developmental delay/mental retardation was included. Studies from Pakistan in which metabolic disorders were evaluated for the markers, technologies and modalities to reach the diagnosis were specifically included. Search was done to find any possible study with published data considering the overall prevalence of metabolic disorders in general population of Pakistan. For the current project unpublished data or data from institutions was not collected at national or individual hospital levels performing CH and IEM related biochemical or genetic tests locally or abroad. Studies, in which probability of IEM was not confirmed by biochemical or molecular testing, were excluded. Data from all studies included in this review was summarized to obtain an overall picture of the selected disease burden from studies based on selected subgroups (Tables 1, 2) and from studies mentioning about various forms of IEM (Table 2).

**Table 1:** Studies related to specific disorders

Diagnosis	Population	Sample size	Age range of presentation	Consanguinity	Genetic MEthods for diagnosis	Biochemical methods for diagnosis	References
Congenital adrenal hyperplasia	Karachi	26 patients	Not mentioned	Not mentioned	Genetic ARMS-PCR (amplified refractory mutation system)	Not mentioned	[18]
Congenital adrenal hyperplasia	Karachi	63	1 day to 12 year	33 cases (52.3%)	Not mentioned	Enzyme assays mentioned	[19]
Congenital adrenal hyperplasia	Karachi	Case series 3 cases	47,20,24 year	positive	Genetic analysis through PCR	Progesterone, testosterone levels done	[20]
Congenital adrenal hyperplasia	AFIP Rawalpindi	Case report	5 years	positive	Not mentioned	Progesterone, testosterone levels done	[21]
Congenital adrenal hyperplasia	AKU Karachi	29 patients	Not mentioned	positive 65%	Mutation analysis done	Progesterone, testosterone levels done	[22]
Lysosomal storage disorder: Gaucher's Disease	Aga Khan University, Karachi, Pakistan, with different forms	2 patients	Not mentioned	Not mentioned	Not Done	BM, Hematological parameters, acid phosphatase level, visceral volumetric CT and MRI, xray, DEXA	[23]
Lysosomal storage disorder: Gaucher's Disease	AKU Karachi	Case report	Not mentioned	Not mentioned	Not Done	Acid phosphatase level done	[24]
Lysosomal storage disease	Peshawar	22 patients Gaucher disease 15 (68%) Niemann- Pick Disease in 7 (30.8%)	Not mentioned	Not mentioned	Not mentioned	A total of 413 bone marrows were aspirated in 2 months	[25]
Gaucher’s Disease	National Institute of Blood Disease and Bone marrow Transplantation	5 patients out of total 19 patients (10 parents 4 control)	Not mentioned	Not mentioned	Identification of GBA Gene Mutations	Β-glucosidase enzyme Levels rather than On bone marrow Morphology	[26]
Lysosomal storage disorder: Gaucher’s Disease Type 1	Civil Hospital, Karachi	Case report	18 months	Not Done	Not Done	Low leukocyte glucocerebrosidase activity, raised plasma chitotriosidase and the presence Of Gaucher cells on bone marrow biopsy. The disease was treated with Intravenous replacement of The enzyme Imiglucerase (cerezyme) and the patient was followed	[27]
Niemann-pick disease	Children’s Hospital Lahore	Total seven sporadic patients	Not mentioned	Unrelated patients from consanguineous families	We have mapped five different mutations in SMPD1 gene of enrolled patients with a novel Homozygous missense variant (c.1718G > C) (p.Trp573Ser) in one patient. A missense mutation (c.1267C > T) (p.His423Tyr) has been identified in three unrelated patients. A nonsense mutation (c.1327C > T) (p.Arg443Term) and one missense mutation (c.1493G > A) (p.Arg498His) mapped in one patient each. A Compound heterozygous mutation has been mapped in one patient (c.740G > A) (p.Gly247Asp); (c.1493G > A) (p.Arg498His). Pathogenic effect of novel variant has been predicted through in-silico analysis and has not Been reported in general overall population in the globe	Reduced acid sphingomyelinase activity in fibroblasts, Lymphoblasts or in peripheral white blood cells	[28]
MPS	KPK, Punjab, Baluchistan, FATA	8 families	Not mentioned	Not mentioned	DNA extraction Sanger sequencing Insilico (QAU) Linkage analysis followed by sequence analysis of the gene detected four novel (p.Phe216Ser, p.Met38Arg, p.Ala291Ser, p.Glu121Argfs*37) and two reported (p.Pro420Arg, p.Arg386Cys) mutations in the eight families. In silico structural and functional analysis predicted that these mutations disrupt the function of GALNS protein through fluctuating its three-dimensional structure, stability, and binding affinity and produce severe phenotypes	Not mentioned	[29]
MPS	Pakistan	Thirteen MPS1-affected children from 12 unrelated cohorts were enrolled	Not mentioned	Not mentioned	Results Six IDUA gene mutations were mapped co-segregating with the recessive pattern of inheritance including a novel variant. A novel missense variant c.908 T > C (p.L303P) was mapped in two affected siblings in a cohort in the homozygous form. The variant c.1469 T > C (p.L490P) was mapped in five unrelated patients and c.784delc (p.H262Tfs*55) was mapped in three unrelated patients, while mutations c.1598C > G (p.P533R), c.314G > A (p.R105Q) and c.1277ins9 [p. (A394-L395-L396)] were mapped in a single patient each	Not mentioned	Mapping of IDUA gene variants in Pakistani patients with mucopolysaccharidosis type 1
							Muhammad Yasir Zahoor, Huma Arshad Cheema, + 3 authors Munir Ahmad Bhinder
							Published in Journal of pediatric…2019
							Medicine
							Journal of Pediatric Endocrinology and Metabolism
Type 1 Galactosemia (Classical and Duarte)	Department of Pediatric Gastroenterology and Hepatology, Children's Hospital and Institute of Child Health, Lahore	8 Families	1.6–15 months	6 Families	Detection of common mutations in the GALT gene through ARMS Done localy	Not mentioned	[13]
Galactosemia	Department Of Pediatric Gastroenterology Hepatology at The Children’s Hospital and Institute of Child Health. Lahore	22 patients	Mean age 112 days with a range from 8—510 days	Not mentioned	Not Done	Benedict’s test (urine), Dipstick (Glysinuria) Enzyme analysis GAL-1 PUT	[30]
GSD Type 1a	Department of Pediatric, division of Gastroenterology & Hepatology of the Children’s hospital, Lahore	40 pts with GSD out of 360 with liver disoder	25.6 months	Not mentioned	Not Done	Clinical and Biochemical test based diagnosis	[31]
Methylmalonic aciduria:	January 2013 to April 2016 at the Aga Khan University Hospital, Karachi	1,778 patients 50(2.81%) were detected with methylmalonic acidurias.	Not mentioned	Not mentioned	Not Done	Methyl melaonic aciduria is a biochemical finding present in patients with MMA, Cb1-RD, SUCL deficiency and serum B12 deficiency thus all patients with mmauria should be further Investigated with PAA, thcy, B12 and FA levels for the correct diagnosis. A correct diagnosis allows clinicians to prescribe appropriate treatment, leading to better Outcome	[32]
Tyrosinemia Type 1 and Fructose-1, 6 Bisphosphatase Deficiency	Pakistani cohorts Children hospital lahore	4 cohorts Hepatorenal tyrosinemia type 1 (HT1) and 8 cohorts fructose 1,6-bisphosphatase deficiency (FBPD	Not mentioned	Not mentioned	Mapping of two recessive mutations in FAH gene for HT1; c.1062 + 5G > A(IVS12 + 5G > A) in three families and c.974C > T(pt325m) in one. We identified three mutations in FBP1 gene; c.841G > A(p.E281K) in five FBPD families, c.472C > T(p.R158W) in two families and c.778G > A(p.G260R) in one	Not mentioned	Genetic Analysis of Tyrosinemia Type 1 and Fructose-1, 6 Bisphosphatase Deficiency Affected Pakistani Cohorts. Muhammad Yasir Zahoor, Huma Ashraf Cheema, Sadaqat Ijaz, Zafar Fayyaz less Published in Fetal and pediatric pathology 2019 Medicine
Alkaptnuria	Mayo hospital lahore	2 Cases	Non-consanguinity	Not Done	Urine analysis HGA	Biochemical assays	Alkaptonuria – case report and REview of literature Muhammad Nafees1, Muhammad Muazzam. Pak J Med Sci 2007 Vol. 23 No. 4 www.pjms.com.pk

**Table 2:** Studies on congenital hypothyroidism

Diagnosis	Population	Sample size	Age range of presentation	Consanguinity	Genetic Methods for diagnosis	Biochemical methods for Diagnosis	References
Congenital hypothyroidism	Karachi	4 out of 5000	Neonate birth to 1 month	Not mentioned	Not Done	Thyroid function tests through immunoradiometric assay	[33]
Congenital hypothyroidism	Karachi	116 hypothyroid 46 with vitamin B12 deficiency	19 year to 91 year	Not mentioned	Not Done	Thyroid function tests	[34]
Congenital hypothyroidism	Karachi	80 hypothyroid 80 normal mothers	1 to 3 month postpartum	Not mentioned	Not mentioned	Thyroid function tests	[35]
Congenital hypothyroidism	Pediatric department PIMS Islamabad	3 babies out of 1337 had CH	Neonates less than 8 days	Not mentioned	Not mentioned	Thyroid function tests through immunoradiometric assay	[36]
Congenital hypothyroidism	Gynae/Obs and Pediatric Shaikh Zayed Hospital and Jinnah Hospital, Lahore	2 out of 1357 cases	neonates	2 patients of CH	Not Mentioned	TSH levels	[37]
Congenital hypothyroidism	Department of pediatrics Mayo hospital lahor	4 out of 550 screened	Neonates 4th–7th day of life	Not mentioned	Not mentioned	Thyroid function tests through immunoradiometric assay	[38]
Congenital hypothyroidism	Pathology Department of Allama Iqbal Medical College, Lahore in collaboration with Pediatrics and Gynecology & Obstetrics Department, Jinnah Hospital, Lahore Pakistan. Lahore	3 hypothyroid out of 770 screened neonates	Not mentioned	Not mentioned	Not mentioned	Serum TSH by immunoassay	[39]
Congenital hypothyroidism	Karachi	400 multiparous women	< 26 to > 35 year	Not mentioned	Not mentioned	TSH screening done survey based	[40]

## Data related to studies

Based on review of these studies data we were be able to include total of 19 IEM specific studies 8 with CH and 11 mentioning about various forms of IEM in a single article. Out of 19 studies related to IEM five were regarding congenital adrenal hyperplasia (CAH) mainly focusing about the diagnosis, research and management based findings. Eight studies were about lysosomal storage disorders, three were about carbohydrate storage disorders including Galactosemia, Glycogen storage disorders, Fructose 1, 6, Bisphosphate. Two studies specifically mentioning about organic acidemia and one about disorders of aminoaciduria. Total numbers of patients mentioned in various studies were found to be around 313. Out of which 78 patients with carbohydrate storage disorder, 66 with lysosomal storage disorder, 47 with organic acidemia and aminoaciduria and 122 with congenital adrenal hyperplasia. In almost all studies biochemical analysis was discussed to reach the diagnosis but significant delay in diagnosis was due to lack of specific diagnostic facilities. Eleven studies specifically mentioned about the use of genetic test for research purpose. Few basic genetic based diagnostic tests done in Pakistan like PCR, RFLP. But sequencing based data mentioned in studies usually obtained from the international labs working in collaboration with academic institutions. None of the study mentioned regarding the availability or use of advance forms of management options like gene editing etc. (Table 1).

8 studies specifically discussed about congenital hypothyroidism. Seven were on biochemical profile of patients and in one survey tool was designed and implemented to collect data from 400 multiparous women. Number of Patients with congenital hypothyroidism tested through biochemical analysis was 212. None of these studies mentioned about genetic tests use and availability for patients with CH (Table 2).

In 11 studies disorders were investigated with the aim to find about various varieties of IEM. Total number of patients were about 536 including 188 patients from various types of carbohydrate storage disorders like Galactosemia, Glycogen storage disorders, 188 with Lysosomal storage disorders including Mucopolysaccharidosis. 220 with organic acidemia, 64 with Aminoacid disorder, alkaptnuria, tyriosinemia. 4 were with congenital adrenal hyperplasia, 7 with FA oxidation and Carnitine defect, 7 with Ketogenesis and ketolytic defect and 19 with various forms of IEM. Biochemical Analysis was done in all while genetic analysis was not available in any study (Table 2).

## Discusssion

According to this data we can say that access to Screening is primarily available at a cost from private laboratories within Pakistan and abroad [5, 10, 11]. Through tandem-MS technology we can simultaneously test for more than 40 disorders. Conventional methods help us to diagnose common disorders like CH, congenital adrenal hyperplasia (CAH) and Galactosemia etc. DNA based testing is not freely available for screening and diagnosis. Few research institutes in Pakistan are working with international collaboration and they are offering genetic tests for few common disorders wherein causal gene is already known [12–17]. But for a proportion of IEM, the causal gene or the mutations in various genes remain to be identified in our ethnic population and thus newborn screening (NBS) program encompasses a strong research component as well [5].

In Pakistan earliest reports of IEM have been far and rare and mostly based on testing newborns with suspicion of IEM or referred to tertiary care centers for intensive care. Some studies included group of subjects who were tested for intellectual disability (Tables 1, 2) [11]. A number of studies from different centers and universities in Pakistan have considered the Pakistani population in the last 15 years for biochemical and genetic analysis and have diagnosed congenital hypothyroidism (CH), condential adrena hyperplasia (CAH), carbohydrate storage disorders, lysosomal storage disorders and organic acidemias (Tables 1, 2). These disorders have been tested at a number of centers, and samples mostly collected from intensive care departments, pediatric units and from hematology units of tertiary care hospitals in Pakistan including Karachi, Lahore, Islamabad, Peshawar and Faisalabad. Mostly patients referred to these centers for diagnosis and better management [5, 12–15] (Tables 1, 2). It is apparent from the data compiled and presented that there is a need for establishing/identifying reliable labs for biochemical testing and genetic confirmation and taken together, warrant formulation of a standard protocol for testing and follow up of IEM positive cases in the country.

In summary, the present findings suggest that expanded NBS for all treatable and untreatable IEM is carried out at a very few centers in the country; NBS for common treatable conditions namely CH and few organic academia are being carried out in a limited number of hospitals at a few places such as Islamabad, Karachi and Faisalabad in pediatric cohorts with index of suspicion of IEM [5, 8, 10, 11]. Patients are tested on referral at several hospitals/private testing labs in the country; but epidemiological data for all the testable or untreatable IEM in Pakistan are not yet available.

## Conclusion:

Keeping the genetic landscape of the Pakistani population (for example diversity) combined with socio-cultural practices (for example consanguinity), the need to carry out systematic prospective expanded screening for IEM in each of the Pakistani province to identify the prevalent disorders is imminent. Finally, ongoing and future prospective studies in Pakistan will be useful with moderate sample size in the country which will provide the much needed epidemiological data for this large group of genetic disorders. In future we must try to overcome challenges of IEM in our country including new born screening (NBS). Findings from these studies from different regions of Pakistan will help Pakistan’s health policy makers to mandate universal and expanded NBS in different provinces in the country.

## Data Availability

With author.

## Abbreviations

IEM: Inborn errors of metabolism
NBS: New born screening
CH: Congenital hypothyroidism
CAH: Congenital adrenal hyperplasia
